# Reconstruction of advanced bone defect associated with severely compromised maxillary anterior teeth in aggressive periodontitis: a case report

**DOI:** 10.1186/s13256-015-0677-6

**Published:** 2015-09-25

**Authors:** Wisam Kamil, Lina Al Bayati, Akbar S. Hussin, Haszelini Hassan

**Affiliations:** 1 Periodontics Unit, Kulliyyah of Dentistry, International Islamic University Malaysia, Kuantan, Pahang Malaysia; 2 Orthodontics Unit, Kulliyyah of Dentistry, International Islamic University Malaysia, Kuantan, Pahang Malaysia; 3 Oral & Maxillofacial Surgery Unit, Kulliyyah of Dentistry, International Islamic University Malaysia, Kuantan, Pahang Malaysia

## Abstract

**Introduction:**

Aggressive periodontitis is characterized by a rapid rate of attachment loss and bone resorption. Regenerative therapy offers reconstruction of the periodontium; however, certain advanced cases with a questionable prognosis might remain a challenge. We report a successful intervention outcome of a challenging case in the aesthetic zone of a patient with aggressive periodontitis.

**Case presentation:**

A 34-year-old systemically healthy Malay woman was referred to the Periodontics Specialist Clinic of the Kulliyyah of Dentistry, International Islamic University Malaysia, with a chief complaint of bleeding gums and mobility of the upper anterior teeth. A diagnosis of localized aggressive periodontitis was made. A thorough non-surgical periodontal treatment was provided, followed by a series of regenerative periodontal surgeries to manage advanced bone defects. A successful treatment outcome with a good prognosis was achieved. Maintenance through the supportive treatment phase showed marked bone gain.

**Conclusions:**

Teeth with severely compromised periodontium of unpredictable prognosis can still be maintained with satisfactory restoration of the function, support, and aesthetics, despite the baseline unpredicted treatment outcome. Proper selection of an advanced periodontal treatment plan can exclude the option of tooth extraction or prosthetic replacement.

## Introduction

Aggressive periodontitis has been defined in the literature as a rapid form of periodontal destruction that develops early in life and affects systemically healthy individuals [1, 2]. The prevalence of this disease in Asian populations is between 0.2% and 1.0% [3]. A distinct radiographic feature of this form of periodontal destruction is the angular bony defects at the first permanent molars and central incisors [4]. Because there is a rapid rate of alveolar bone resorption in this disease, the circumferential bone defects may progress to more than two-thirds of the roots, affecting the tooth-supporting tissues within a few years, and, as a consequence of disease progression, a high grade of mobility, deep intra-bony defects, and pus discharge can be observed at the involved sites [5].

The relative effectiveness of non-surgical anti-infective periodontal therapy on aggressive periodontitis compared with chronic periodontitis is still unclear [6]. There is a limited opportunity to eradicate the periodontopathogens in the deep sites through non-surgical periodontal therapy (NPT) [7], and the expected healing process in patients treated with non-surgical therapy is by long junctional epithelium rather than through the regeneration, resulting in persistence of bleeding sites and disease recurrence [8]. However, recent investigations revealed the persistence of the periodontal pathogens even after a full mouth extraction [9]. Therefore, regenerative periodontal therapy for advanced periodontal defects to improve the prognosis and longevity of teeth appears promising [10].

Studies of the prognostic model of periodontally compromised teeth [11–13] showed that teeth with >50% bone loss have a questionable prognosis and ultimately “hopeless” if they have inadequate attachment to maintain health. Advanced bone defects, deep pockets, and tooth mobility are found to be associated with increased risk of tooth loss. Moreover, the patient’s condition will be more critical when central incisors in the aesthetic zone are affected, owing to the difficulty in satisfying patient expectations, especially in young women, when choosing the option of tooth extraction. In a recent study [14], Cortellini and co-workers demonstrated the potential effect of regenerative therapy in changing the prognosis of hopeless teeth instead of making a decision to extract such periodontally compromised teeth. However, the inclusion criterion that had been applied included intra-bony defect presenting with a clearly detectable bone crest at the neighboring tooth or teeth.

In this report, we describe a successful clinical outcome in the reconstruction of a circumferential bony defect extending from tooth 12 to tooth 22 over the palatal aspects of the upper central incisors (UCI) of a patient with aggressive periodontitis. This condition was unpredictable and was seen as a challenge, particularly in the aesthetic zone.

## Case presentation

**Fig. 1:**
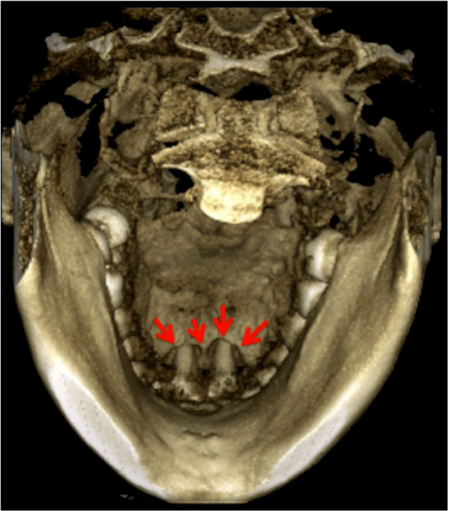
Three-dimensional image displays the circumferential bone defect from the palatal aspect (*red arrows*)

**Fig. 2:**
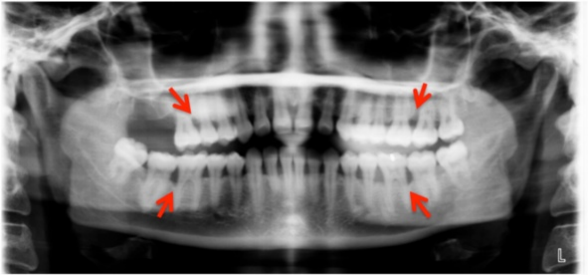
Baseline orthopantomography. *Red arrows* indicate the angular bone defects around the first and/or second molars

A 34-year-old systemically healthy Malay woman was referred to the Periodontics Specialist Clinic of the Kulliyyah of Dentistry, International Islamic University Malaysia, with a chief complaint of bleeding gums and mobility of her upper anterior teeth. Complete medical and dental histories were taken, and a full periodontal chart was recorded, including all the clinical periodontal parameters, plaque scores and bleeding on probing (BOP), probing pocket depth (PPD), clinical attachment loss (CAL), recession, mobility, and furcation involvement. The chart revealed grade II mobility of the UCI and pus discharge with deep probing depths and severe CAL >6mm. The three-dimensional picture in Fig. 1 shows the circumferential bone resorption surrounding the UCI. The rapid rate of bone destruction affected the sites of the first and second molars, resulting in furcation involvement and mobility ranging from grade I to grade II with recorded PD and CAL greater than 6mm as well. The radiographic interpretation showed an angular bone defect mesial and/or distal to the first molars (Fig. 2). Because the patient was systemically healthy except for the presence of periodontitis, and based on the patient’s history, clinical periodontal records, and radiographic findings, she was diagnosed with localized aggressive periodontitis [15]. A combination of oral metronidazole 400mg and amoxicillin 500mg were prescribed for 7 days as an adjunct to scaling and root planing (SRP). Six weeks after the NPT, the affected sites were reevaluated. The results revealed slight reductions in the patient’s BOP, PD, and CAL. However, the range of residual bleeding pocket depths was from 5mm to 6mm with no obvious clinical attachment gain. Therefore, the decision was made to provide regenerative therapy, and a surgical treatment was scheduled for the patient in spite of the questionable prognosis that had been assigned at baseline. The patient was informed verbally regarding the details of the surgical treatment plan and signed a written informed consent form before each periodontal surgical procedure as part of the clinical protocol of our institute.

**Fig. 3:**
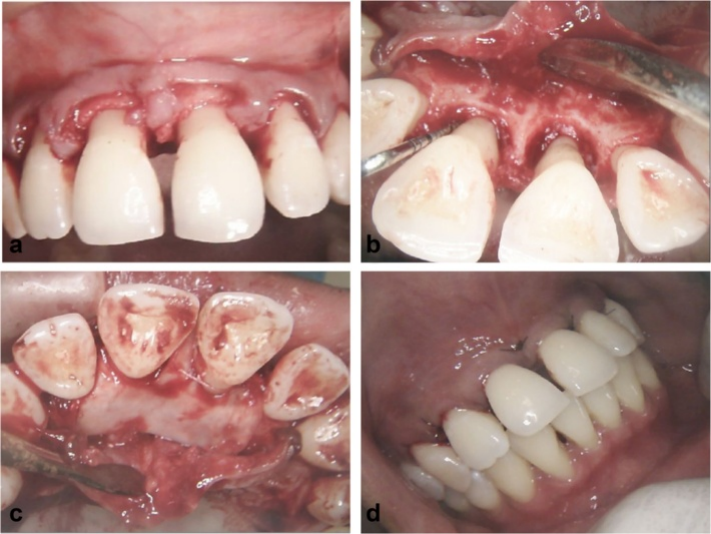
Intra-oral photographs show the surgical area at the upper anterior teeth. **a** Papilla preservation technique illustrating the whole inter-dental papilla with the labial flap. **b** The circumferential bone defect around the upper central incisors after debridement. **c** The surgical area from the palatal aspect highlights the collagen barrier membrane applied to cover the bone graft. **d** Sutures are applied to close the flap

The surgical procedure on the UCI included the use of a papilla preservation technique, which was applied to preserve the papilla and obtain primary closure of the inter-dental space (Fig. 3a). The technique of papilla preservation [16] that we applied, compared with the modified technique [17], provides better access to the palatal defect because it is applicable on the buccal and palatal aspects. Therefore, the incorporation of papillae with the facial part of the flap (Fig. 3a) was achieved through crevicular incisions made around each tooth without splitting the inter-dental papilla, and a semi-lunar incision was made across the palatal aspect of at least 5mm from the papillary crest, taking advantage of the keratinized palatal mucosa in protecting the graft during healing. Additionally, this palatal approach overcomes the post-operative labial scar formation in the aesthetic zone.

**Fig. 4:**
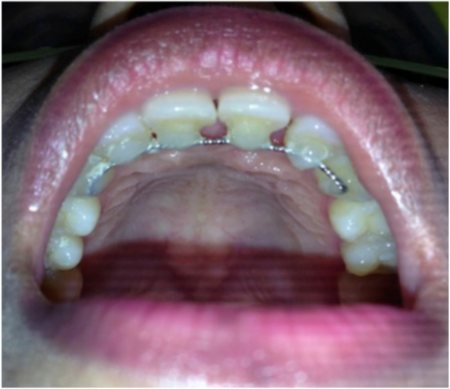
Intra-oral photograph of the palatal aspect shows the splinting of the upper anterior teeth with a fixed retainer and twisted wire

As a result of the intra-bony defect location, which is associated with two adjacent central incisors, significant supra-crestal components of the defect presented at the palatal area (Fig. 1). The circumferential intra-bony defects were thoroughly debrided (Fig. 3b), and cerabone bovine xenograft granules (botiss biomaterials, Zossen, Germany) were used to fill the defects. An OsseoGuard resorbable bovine collagen membrane (Collagen Matrix/BIOMET *3i*, Palm Beach Gardens, FL, USA) covered the bone substitute, with delicate trimming of the membrane done to extend inter-proximally (Fig. 3c) using sling sutures of absorbable material to adapt the membrane well and prevent its collapse over the bone graft. The flap was sutured using an internal horizontal mattress suture, which has the advantage of providing a tension-free flap (Fig. 3d) and a stable support for the grafting area. Simple interrupted sutures were applied at the rest of the flap sites. The sutures were removed 10 days after the surgical procedure. A fixed retainer using twisted wire (177.8mm; 3M Unitek, Loughborough, UK) was constructed palatally to splint the anterior teeth and stabilize the wound healing (Fig. 4). This type of wire allows long-term stability with physiologic tooth movement.

Strict oral hygiene instructions were given; however, the patient was asked to avoid normal brushing and flossing in the treated area for 4–6 weeks and replace it with the use of 0.12% chlorhexidine mouthwash. The modified Stillman brushing method was recommended to prevent the interference beneath the gingival margin until complete resorption of the membrane was achieved. During the healing process, professional plaque control was scheduled every 2 weeks to prevent bacterial contamination of the surgical area. The patient was also instructed not to chew on the treated area for the first 4 weeks.

**Fig. 5:**
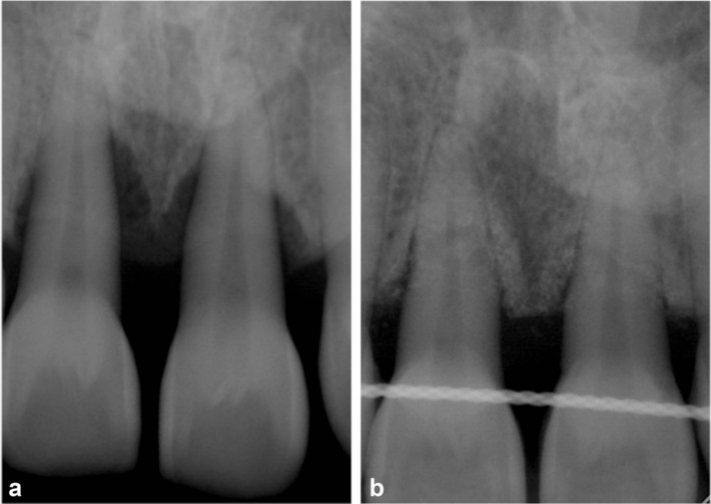
**a** Pre-treatment peri-apical radiograph of the patient’s upper central incisors with circumferential bone resorption. **b** Peri-apical radiograph of the same region 2 years later shows significant bone fill

**Fig. 6:**
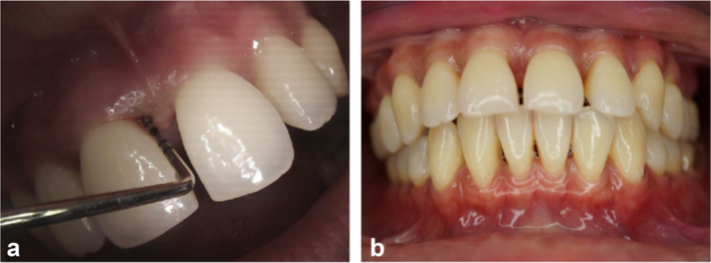
**a** Deep probing depth at the upper central incisors with bleeding on probing and inflamed gingival tissue. **b** Frontal intra-oral photograph shows healthy, firm, non-edematous gingival tissue

The application of the graft and barrier membrane resulted in remarkable bone gain and support for more than two-thirds of the root length of the UCI, as shown in the radiograph taken at 2 years (Fig. 5b), which confirmed the resolution of the defect present at baseline (Fig. 5a). There was absence of BOP, significant PD reduction up to 2mm, and greater than 5mm attachment gain. Additionally, there was almost an equivalent amount of attachment gain and bone fill. The intra-oral photographs in Fig. 6 show the clinical improvement in the gingival tissue from edematous bleeding gingiva (Fig. 6a) to the closely adapted, firm, non-bleeding sites (Fig. 6b). Furthermore, Fig. 6b depicts a good post-operative result without any inter-proximally soft tissue crater formation, which explains the pre-requisite of the papillary preservation technique.

**Fig. 7:**
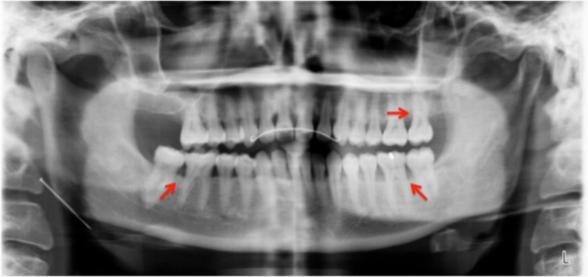
Orthopantomography was performed 2 years after baseline. *Red arrows* indicate area of previous angular bone defects with marked bone gain compared with baseline as indicated in Fig. 2

Three more regenerative periodontal surgeries were performed for regions of teeth 46, 26, 27, and 36. In these procedures, we used different xenograft bone granules of bovine origin (Endobone from Collagen Matrix/BIOMET *3i* and Bio-Oss from Geistlich Biomaterials, Wolhusen, Switzerland) and achieved successful results in respect to bone fill (Fig. 7) compared with baseline (Fig. 2). The patient was covered with systemic antibiotics for all periodontal regenerative surgical procedures. A decision was made to extract the partially erupted and angulated third molars because these teeth were either without antagonists or compromising the adjacent second molars. They also presented difficulties in self-performed plaque control and acted as a local factor for plaque accumulation in deep periodontal pockets. The surgical extractions were performed during the surgical periodontal therapy to minimize the required treatment time and healing process.

During the phase of supportive periodontal therapy, the patient was treated with a schedule of professional plaque control with adjunct use of photodynamic therapy (PDT). The recall system of the maintenance phase was arranged every 3 months. At 2 years after baseline, the full periodontal chart revealed a marked improvement in the clinical periodontal records, as the percentage of sites with BOP had decreased from 40% to 16% and there was a substantial decrease in the number of deep pockets, characterized by the absence of PPD greater than 4mm compared with 22.22% before the commencement of treatment. This PPD reduction was accompanied by an average CAL gain of 3.07mm.

## Discussion

Despite the rapid rate of attachment loss in patients with aggressive periodontitis, the treatment of such cases is not different from that of chronic periodontitis in respect to all phases of treatment [6]. In a recent consensus report by our group [18], it was stated that adjunctive use of systemic antibiotics and PDT supplementary to SRP are anti-infective non-surgical approaches that are required to eradicate periodontopathogens from infected sites. Nevertheless, regenerative surgical treatment is a requisite to restoring the periodontium and ensuring long-term tooth stability [6]. Although the treatment option of teeth with a “hopeless” prognosis was tooth extraction [12], advanced reconstructive therapy and selected surgical techniques could alter the prognosis with an expectation of good results [14].

The clinical presentation of aggressive periodontitis usually includes the involvement of central incisors with angular bone defects [4]. Therefore, it is fundamental to devise a good treatment plan to retain these adjacent teeth rather than extract them. Similarly, it is difficult to establish the periodontal papilla between two neighboring implants [19]. In a recent consensus report of the American Academy of Periodontology regeneration workshop [20], its authors concluded that early intervention for intra-bony defects with regenerative approaches can be successful. In respect to the treatment of patients with aggressive periodontitis with bone grafting materials, our review of the literature revealed few published case reports [21–23] and controlled studies [24, 25]. Nevertheless, these cases were restricted merely to separated angular defects around premolar or molar teeth compared with the extent of defects affecting the maxillary two central incisors in our patient.

Although the immobilization of highly mobile teeth through periodontal splinting does not offer a successful periodontal improvement, stabilization of teeth during the regenerative treatment is important to stabilizing the wound healing [18]. The splinting that we applied was considered to be permanent for the patient to protect the newly gained bone from the occlusion force in the long term, as the bone substitute we used was osteoconductive in nature.

A well-established plaque control regimen with excellent patient compliance in aggressive periodontitis cases is highly recommended in the maintenance phase to ensure good periodontal health [6]. Therefore, professional and self-performed plaque control measures were instituted to help the patient achieve good oral hygiene.

## Conclusions

Periodontally compromised teeth with unpredictable prognosis in the aesthetic zone of patients with aggressive periodontitis can be managed through a comprehensive treatment plan that includes an advanced surgical reconstruction approach to achieve a favorable long-term prognosis, maintain the natural healthy dentition, and overcome the need for prosthesis.

## Consent

Written informed consent was obtained from the patient for publication of this case report and any accompanying images. A copy of the written consent is available for review by the Editor-in-Chief of this journal.

## Abbreviations

BOP: bleeding on probing
CAL: clinical attachment loss
NPT: non-surgical periodontal therapy
PDT: photodynamic therapy
PPD: probing pocket depth
SRP: scaling and root planing
UCI: upper central incisors
